# The complex role of IL-37 in cancer pathogenesis: exploring its influence on hallmarks of cancer

**DOI:** 10.3389/fimmu.2026.1738156

**Published:** 2026-05-19

**Authors:** Stavros P. Papadakos, Ioanna E. Stergiou, Maria S. Andreou, Ioannis Stouras, Elena Chatzikalil, Maria Tzimou, Christina Karantanou, Maria-Ioanna Christodoulou, Stamatios Theocharis

**Affiliations:** 1 First Department of Pathology, Medical School, National and Kapodistrian University of Athens, Athens, Greece; 2 Department of Pathophysiology, School of Medicine, National and Kapodistrian University of Athens, Athens, Greece; 3 Tumor Immunology and Biomarkers Laboratory, Basic and Translational Cancer Research Center, Department of Life Sciences, School of Sciences, European University Cyprus., Nicosia, Cyprus; 4 Department of Medicine, School of Medicine, European University Cyprus, Frankfurt am Main, Germany

**Keywords:** cancer pathogenesis, hallmarks of cancer, IL-37, immunotherapy, inflammation

## Abstract

Interleukin-37 (IL-37) is a cytokine primarily known for its anti-inflammatory properties, but its role in cancer pathogenesis reveals a complex and multifaceted influence. This review explores IL-37’s diverse effects on fundamental hallmarks of cancer, such as tumor cell proliferation, invasion, metastasis, and interactions with the immune system. We discuss how IL-37 can modulate these processes in different contexts—sometimes impeding tumor growth and other times facilitating it—depending on factors including the tumor type, stage, and surrounding microenvironment. The dual nature of IL-37’s influence underscores its potential as both a therapeutic target and a biomarker for cancer progression, which remain to be validated in prospective clinical studies. By integrating recent findings, this review aims to provide a comprehensive understanding of IL-37’s contributions to cancer biology, offering insights into its possible future applications in personalized cancer treatment strategies and enhancing our ability to tailor interventions based on the specific roles of IL-37 in individual cancers.

## 1 Introduction

Human cancer is considered the leading cause of death and treatment-related morbidity worldwide (1). Drastic improvements in survivorship have been achieved, with encouraging results in patients’ clinical outcomes for some diagnoses (e.g., ALL, Hodgkin lymphoma). However, other types remain intractable (e.g., metastatic HCC), characterized by a plateau in patients’ survival after years of using the available therapies in cases with recurrent disease (2). A series of novel anti-tumor agents are currently used in clinical trials or preclinical models, as potential weapons in the fields of precision and personalized medicine (3–5). However, limited clinical evidence related to the efficacy of many of them in certain oncologic diagnoses and in special populations (e.g., pediatric patients) currently exist, due to the rarity of the first (<1/10.000 in the general population for some of them), and to the exclusion of the second from established treatment indications or from late phase clinical trials (6). As a result, the need for continuous research on novel anti-tumor agents with a well-studied therapeutic effect and safety profile in many tumor types, is mandatory, especially for neoplasms with high recurrence and metastasis rates (7).

Cancer progression involves the acquisition of key biological traits, or “hallmarks,” that enable tumor cells to proliferate uncontrollably, evade cell death, induce angiogenesis, invade tissues, metastasize, maintain genomic instability, promote inflammation, reprogram metabolism, and escape immune surveillance (8–13). Importantly, these hallmarks are not autonomous tumor cell–intrinsic events but are dynamically shaped by cytokine-driven signaling networks within the tumor microenvironment. Inflammatory and immunoregulatory pathways converging on central signaling hubs such as NF-κB, STAT3, and Smad3 play pivotal roles in coordinating hallmark-associated processes, including proliferation, survival, angiogenesis, immune tolerance, and metabolic adaptation. Consequently, cytokines capable of modulating these pathways may exert pleiotropic and context-dependent effects on cancer development and progression (PMID: 29247987). Within this framework, the Hallmarks of Cancer provide a biologically meaningful context through which the varied functions of IL-37 can be examined.

Although IL-37 has been extensively investigated as an anti-inflammatory and immunoregulatory cytokine across a broad range of human diseases, its role in cancer biology has not been systematically conceptualized within an established oncologic framework (PMID: 34248996). Previous reviews have primarily approached IL-37 from a systemic immunology perspective, in which cancer is discussed alongside autoimmune, metabolic, and infectious conditions (PMID: 29247987). In contrast, the present work adopts a cancer-centered perspective and was designed as a narrative (scoping) review, aiming to integrate IL-37–associated signaling pathways into the Hallmarks of Cancer paradigm and to examine their context-dependent effects on tumor cell behavior, immune regulation, and tumor–microenvironment (TME) interactions.

The literature search was performed in the PubMed database to identify relevant studies published up to September 2025. The terms “IL-37” or “interleukin-37” in combination with cancer-related keywords, including those associated with established cancer hallmarks, were used in the search strategy. Inclusion criteria included *in vitro*, *in vivo* or human observational studies investigating the role of IL-37 in cancer. Exclusion criteria included articles not published in English and those without accessible full text. Given the narrative nature of this review, no formal systematic review framework, quantitative synthesis, or risk-of-bias assessment was applied. Instead, the aim was to provide a structured and integrative overview of current preclinical and clinical evidence regarding IL-37 in cancer, with an emphasis on its potential biological and translational significance, while acknowledging that many findings remain to be validated in clinical settings.

## 2 Structure, signaling and regulatory pathways

Interleukin-37 (IL-37) represents a widely investigated cytokine in terms of structure and functionality due to its involvement in various biological activities and its interaction with a large number of transcription factors, including NFκB and AP1 (14, 15). Preclinical studies by Taylor et. al, described “FIL1-zeta”, a cDNA encoding IL1F7, experimentally validated to be expressed in immune tissues, placenta, lung, and testis as well as in multiple hematopoietic cell lines (15, 16),. Similarly, Kumar et. al, by computational sequence analysis for IL-1 homologs screening identified the same cDNA, which they named “IL1H4” (17). In 2001, IL-1H4 was replaced by the term “IL-1F7”, due to the identification of the seventh cytokine of the IL-1 family as its precursor peptide (14). The term interleukin-37 (IL-37) was established by Nold et. al, who investigated its role as an innate immunity suppressor (14). The human IL-37 gene cluster (IL-37) is located at 2q12-q14.1 on chromosome 2, sized 3.617 kb, and is composed of six exons, which encode a 17–26 kDa protein product (18). IL-37 is an immature precursor peptide in inactive state which turns into active state via its cleavage by caspase-1, between D20 and E21 amino acid residues (18). IL-37 expression is regulated by several stimuli. It is induced by pro-inflammatory signals and pathogen-associated molecular patterns, including TLR ligands such as LPS, Pam3CSK4, and CpG, and by cytokines such as IL-1β, IL-18, TNF, IFN-γ, and TGF-β1 (14). Conversely, IL-4 in combination with GM-CSF downregulates or inhibits its expression (14).

Five basic subtypes of IL-37 have yet been described, as a result of structural alterations via alternative splicing, termed IL-37a, IL-37b, IL-37c, IL-37d, and IL-37e, which are encoded by different exons combinations: exons 3, 4, 5, and 6 encode IL-37a (17–25 kDa), exons 1,2,4,5 and 6 encode IL-37b (21.55 kDa), exons 1, 2, 5, and 6 encode IL-37c (19.61 kDa), exons 1, 4, and 6 encode IL-37d (21.95 kDa), and exons 1, 5, and 6 encode IL-37e (17.46 kDa) (19). IL-37b, consisting of exons 1,2,4,5, and 6, is the largest (218 amino acids) and best described subtype, presenting a β-barrel structure and being characterized by of a N-terminal pre-domain, which consists of a potential caspase-1 cleavage site, and 12 β-strands, which participate in the IL-1-like β-trefoil secondary structure formation (20). IL-37a differs from the other isoforms by a unique start codon in exon 3 which encodes a distinct N-terminus, resulting in a pre-domain which forms the mature form of IL-37a (20). IL-37c, consisting of exons 1, 2, 5, and 6, despite being similar to IL-37b in terms of structural characteristics, is incapable of forming a functional cytokine product (21). IL-37d consists of exons 1 and 4–6 and is capable of producing a normal cytokine product. IL-37e consists of exons 1, 5 and 6 and cannot bind to IL-18R due to the lack of exon 4 (22).

The lack of exon 4 is considered the main reason behind the inability of IL-37c and IL-37e to form protein products with biological functionality, possibly due to the absence of β-clover secondary structure. However, despite IL-37c lack of biological functions, it exhibits an important role in IL-37b and IL-37d expression, specifically by downregulating them, by targeting the same cleavage caspase-1 site (23). Similarly, IL-37e shares the same enzyme cleavage target with IL-37b but does not compete with IL-37d due to deletion of exon 2 in the second cleavage site. As a result, the mature active form of IL-37b can be fully downregulated by IL-37c and partially downregulated by IL-37e (23). The expression of the distinct subtypes of IL-37 is tissue specific. IL-37a, b and c are mainly expressed in thymus, bone marrow, lymph nodes, liver, lung, testis, placenta, uterus, colon, NK cells, monocytes, stimulated B cells, and keratinocytes, while IL-37d and IL-37e are expressed only in bone marrow and testis. IL-37a and IL-37b are also expressed in human PBMCs and cell lines including A431, THP-1, U937, IMTLH, KG-1, HL60, HPBMC, HPT-4, RAJI, SK-LU-1, CCL-247 and NHDC, and THP-1, U937, IMTLH, HL60, and HPT-4, respectively (23).

IL-37 is initially produced in an immature form without a signal peptide and exists in the cytoplasm and nucleus. Upon activation by inflammatory stimuli, caspase-1 cleaves IL-37 into an active form (19, 24, 25). This mature IL-37 can act through three distinct mechanisms.

**Figure 1:**
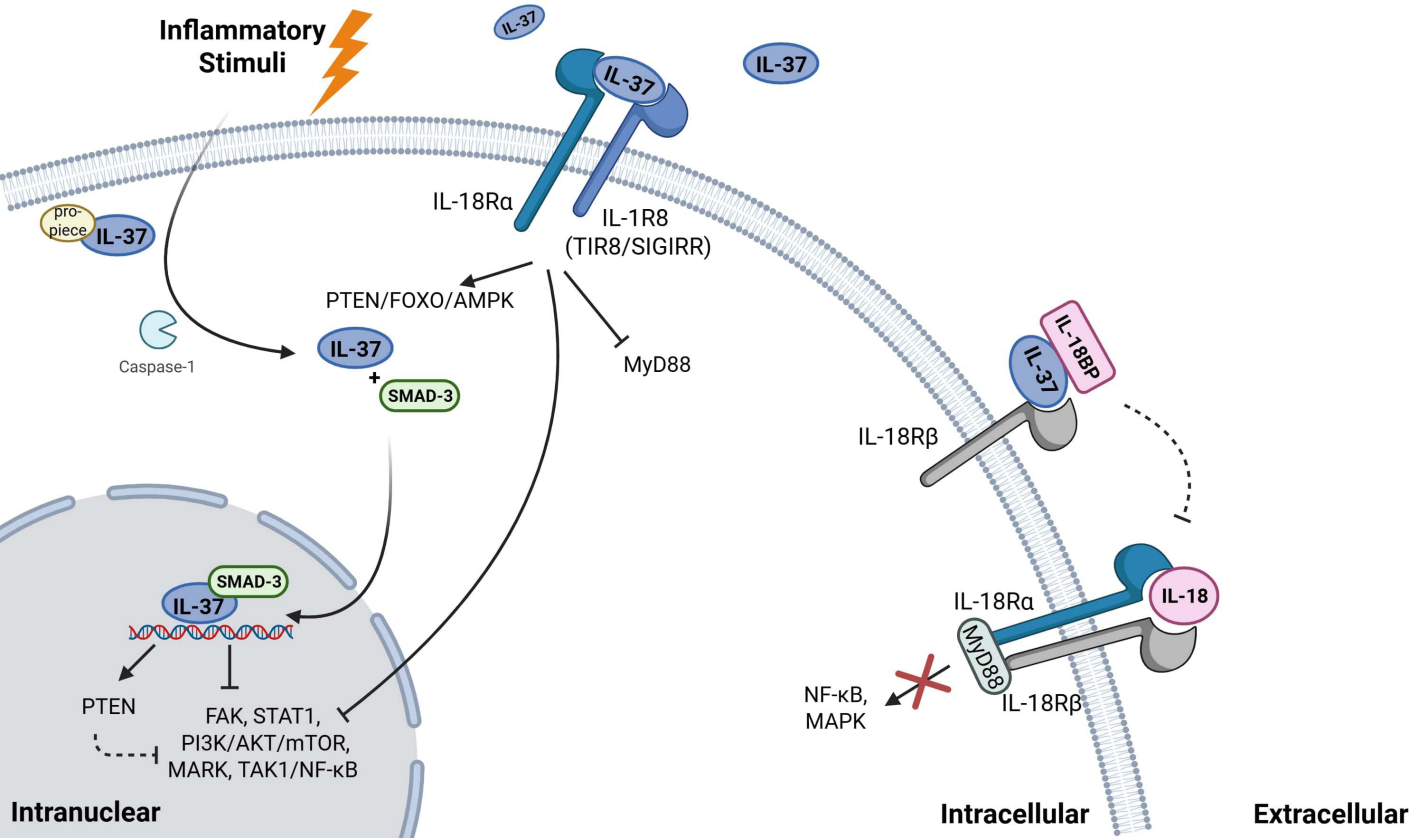
Mechanisms of IL-37 signaling. Intracellularly, IL-37 matures when cleaved by caspase-1. Mature IL-37 will bind to SMAD-3 forming an IL-37/SMAD-3 complex, which can translocate into the nucleus. This translocation will regulate expression of certain genes. Extracellularly, IL-37 binds and forms a triple complex with IL-18Rα and IL-1R8, that exerts the extracellular anti-inflammatory function of IL-37. Extracellular IL-37 can also bind to IL-18BP, forming the IL-37/IL-18BP/IL-18Rβ complex, inhibiting the IL-18 activity. Created by BioRender https://BioRender.com/gupbran.

- Intracellular IL-37: Cleaved IL-37 binds with phosphorylated SMAD-3 forming a complex that translocates into the nucleus (24, 26, 27). This intracellular complex suppresses transcription of pro-inflammatory genes and contributes to the anti-inflammatory effects of IL-37.
- Extracellular IL-37: IL-37 binds with IL-18 receptor α (IL-18Rα, or IL-1R5) and recruits IL-1 receptor 8 (IL-1R8, or TIR8/SIGIRR), instead of IL-18Rβ. This receptor complex is primarily expressed on peripheral blood mononuclear cells (PBMCs) including dendritic cells (DCs) and macrophages. The resulting signaling inhibits the pro-inflammatory pathway myeloid differentiation factor88 (MyD88) (28) and modulates downstream pathways such as PTEN/FOXO/AMPK, MER/DOK(p62), TAK-1/NF-κB, mTOR/FYN and PI3K/AKT/mTOR (29–34). During this process, the mature form of IL-37 demonstrates a significantly higher binding efficiency to the receptor compared to its precursor form. In addition, the production and maturation of IL-1β via the activated factor NLRP3 and Absent In Melanoma 2 (AIM2) inflammasome, are also considered contributors of IL-37 effects (35).
- Extracellular IL-37 and IL-18BP interaction: IL-37 is capable of binding IL-18 binding protein (IL-18BP) (33, 36). As a result, the IL-37/IL-18BP/IL-18Rβ complex is formed, thereby preventing the formation of the functional IL-18R heterodimer (IL-18Rα/IL-18Rβ) and inhibiting the IL-18 activity. However, it has been observed that an excessive binding of IL-18BP to IL-37 may diminish the anti-inflammatory activity of both IL-37 and IL-18BP (34, 37–39). IL-37 signaling pathway is illustrated in **Figure 1**.

IL-37 is expressed in a variety of human tissues, though in low levels because of the unstable and short-half-life mRNA (26), rendering it also an easy target for degradation (23) However, IL-37 expression can be dramatically increased by pro-inflammatory stimulation in several tissues. This includes several toll-like receptor (TLR) agonists, IL-18, interferon (IFN)γ, IL-1b, transforming growth factor β1 and tumor necrosis factor (TNF), and granulocyte-macrophage colony-stimulating factor (GM-CSF) plus IL-4; acting synergistically or not. After stimulation, immune cells (monocytes, dendritic cells, T cells) present higher IL-37 expression levels (14). The stability of IL-37 mRNA may be enhanced by lipopolysaccharide (LPS) or other exogenous stimulation on exon 5 (40). Recent investigation has identified MAPK and pI3K as regulators of IL-37 expression; specifically, ERK1/2 and p38 block IL-37 upregulation caused by the two active Tripterygium wilfordii Hook F herb components (41). Moreover, TNF-α downregulates IL-37b mRNA expression via MAPK and PI3K activation. Mannose-capped lipoarabinomannan purified from Mycobacterium tuberculosis upregulates TLR2 expression and p38 and ERK1/2 phosphorylation, inducing IL-37 expression (42). Other TLR members, like TLR5, are potential enhancers of IL-37 expression (43).

## 3 The role of IL-37 in cancer progression

### 3.1 The influence of IL-37 in proliferation pathways in cancer

IL-37 is a recently identified cytokine with emerging significance in cancer research due to its complex role in modulating immune responses and cellular behaviors (44). Most evidence linking IL-37 to cancer cell proliferation derives from *in vitro* experiments and murine models, with limited direct validation in human cohorts. As a member of the IL-1 family, IL-37 has been primarily recognized for its anti-inflammatory properties. However, its influence extends beyond inflammation, particularly in the realm of cancer biology where it intersects with key proliferation pathways (44). Recent studies suggest that IL-37 may affect tumor growth and progression by altering cellular proliferation dynamics (45). Although current evidence is predominantly preclinical, understanding how IL-37 interacts with these pathways could provide insights into novel mechanisms of tumor regulation and suggest potential therapeutic targets.

Jiang et al. investigated the role of IL-37 in renal cell carcinoma (RCC) (46). They found that IL-37 levels were significantly lower in RCC patients compared to healthy controls, and these reduced levels were associated with more advanced stages of the disease. To explore this association further, the researchers conducted *in vitro* experiments using two RCC cell lines, A498 and Caki-1. They demonstrated that IL-37 inhibited cell proliferation and migration while promoting apoptosis in these cancer cells, in a dose-dependent manner. They identified that IL-37’s antitumor activity was mediated through the suppression of the IL-6/STAT3 signaling pathway. IL-37 reduced IL-6 levels, leading to decreased activation of STAT3 and downregulation of key downstream proteins such as Bcl-2, cyclin D1, and HIF-1α. In addition to the *in vitro* findings, *in vivo* experiments with RCC mouse models showed that IL-37 treatment resulted in significant tumor growth inhibition. The treatment also led to lower levels of IL-6 and HIF-1α within the tumors, further supporting IL-37’s role as a tumor suppressor (46). This study suggests that IL-37 exerts tumor-suppressive effects in RCC models, supporting its potential relevance as a therapeutic target pending validation in clinical settings. Analogously, Wang et al. explored the role of IL-37 in cervical cancer (CC), focusing on its ability to suppress tumor cell proliferation and invasion by inhibiting the STAT3 signaling pathway (47). They utilized both HPV-positive Hela cells and HPV-negative C33A cells, where IL-37 was introduced through gene transfection. The results demonstrated that IL-37 significantly reduced the proliferation and invasion capabilities of these cancer cells, with a stronger effect observed in HPV-positive Hela cells (47). Mechanistically, they found that IL-37 inhibited the expression and phosphorylation of STAT3 at both the mRNA and protein levels. Further experiments showed that silencing STAT3 diminished the inhibitory effects of IL-37 on cancer cell growth and inflammatory cytokine production, such as TNF-α and IL-1β. Conversely, overexpression of STAT3 counteracted the suppressive effects of IL-37, restoring cell proliferation, invasion, and cytokine expression. The findings suggest that IL-37 has significant anticancer properties in CC, primarily through its interaction with the STAT3 pathway (47). This study suggests that IL-37 may have potential relevance in cervical cancer, although its therapeutic applicability remains to be validated in clinical settings. In a study of Zhang et al., that will be in depth analyzed elsewhere, the therapeutic potential of a vaccinia virus engineered to express IL-37 (VV-IL-37) in hepatocellular carcinoma (HCC) was explored (48). They found that VV-IL-37 effectively inhibited the proliferation of HCC cells by reducing STAT3 phosphorylation. *In vivo* experiments showed that VV-IL-37 significantly suppressed tumor growth by promoting antitumor immune responses. The findings suggest that VV-IL-37 represents a potential gene-therapy approach in preclinical HCC models, warranting further investigation before clinical translation. Additionally, Liu et al. explored the role of the IL-37 in HCC (49). They found that IL-37 is typically downregulated in HCC tissues and cell lines compared to non-cancerous liver tissues. This reduced expression of IL-37 is associated with larger tumor size, increased vascular invasion, and poorer overall survival (OS) and disease-free survival (DFS) in patients. Further investigation revealed that IL-37 plays a tumor-suppressive role by causing cell cycle arrest at the G2/M phase, thereby inhibiting tumor growth both *in vitro* and *in vivo*. Mechanistically, IL-37 shifts Smad3 signaling pathway from a cancer-promoting state (JNK/pSmad3L/c-Myc) to a tumor-suppressive state (pSmad3C/p21). They also identified a significant inverse relationship between IL-37 expression and the oncogenic pSmad3L levels in HCC tissues, suggesting that high levels of IL-37 could be a favorable prognostic marker for HCC (49). Overall, the findings underscore the potential of IL-37 as a biomarker for prognosis and a therapeutic target in HCC, highlighting its role in modulating key signaling pathways that influence tumor progression.

Yan et al. investigated the role of IL-37 in colorectal cancer (CRC), emphasizing its potential as an anti-tumor agent (50). To explore this, they analyzed IL-37 expression in 186 pairs of colon cancer tissues and their adjacent normal tissues. The results showed that IL-37 expression was significantly reduced in cancerous tissues compared to the adjacent non-cancerous ones. This reduction in IL-37 expression was closely associated with more advanced stages of cancer, including deeper invasion, lymph node involvement, and distant metastasis. Moreover, lower levels of IL-37 correlated with poorer survival rates, indicating that IL-37 could serve as an independent prognostic marker for CRC. They further revealed that IL-37 had a strong inhibitory effect on colon cancer cell behavior. When IL-37 was overexpressed in CRC cells, it significantly reduced their ability to proliferate, migrate, and invade, while also decreasing the number of cancer stem cells. These anti-tumor effects were mediated through the suppression of the β-catenin signaling pathway. In addition to the *in vitro* findings, they extended this investigation to an *in vivo* mouse model. Therein, the overexpression of IL-37 led to a marked reduction in tumor size and number. Furthermore, IL-37 increased the sensitivity of cancer cells to commonly used chemotherapeutic drugs, suggesting that it may help overcome drug resistance, a significant challenge in cancer treatment. They concluded that IL-37 plays a vital inhibitory role in colon cancer development by suppressing β-catenin expression and activity (50). This suppression hinders the aggressive behavior of cancer cells, supporting its potential relevance as a target for future therapeutic investigation.

Mu et al. investigated the role of IL-37 in suppressing lung adenocarcinoma growth, specifically focusing on its influence on m6A RNA methylation (51). M6A, or N6-methyladenosine, is a prevalent RNA modification involved in the regulation of gene expression and has been implicated in cancer development (52). They explored how IL-37 affects m6A methylation patterns in human lung adenocarcinoma cells (A549 cells) and its potential mechanisms in reducing tumor proliferation. They used high-throughput sequencing methods, such as m6A-specific RNA immunoprecipitation (MeRIP-seq), to analyze the differences in m6A modification patterns between IL-37-treated cells and untreated controls. The study found that IL-37 treatment led to significant changes in m6A methylation levels, particularly influencing the expression of key m6A “writers” (like METTL3, METTL14, and WTAP) and “erasers” (such as ALKBH5 and FTO). These changes in m6A methylation were associated with the downregulation of the Wnt5a/5b signaling pathway, which is crucial for cancer cell proliferation. The findings suggest that IL-37 exerts its anti-tumor effects by modulating m6A methylation, thereby inhibiting pathways that promote cancer cell growth (51). This mechanism highlights IL-37’s potential as a therapeutic agent in treating lung adenocarcinoma and possibly other cancers. Finally, Chen et al. investigated the effects of CCL22 and IL-37 on the proliferation and epithelial-mesenchymal transition (EMT) of A549 cells, a model for non-small cell lung cancer (NSCLC) (53). The results revealed that both CCL22 and IL-37 individually suppressed the proliferation of A549 cells compared to controls and blank plasmid groups. Notably, the combination of both cytokines led to an even more pronounced reduction in cell proliferation than the cytokine alone, indicating a possible additive or synergistic effect. These findings suggest that CCL22 and IL-37 interfere with tumor cell growth, potentially offering therapeutic benefit in NSCLC. Additionally, CCL22 and IL-37 suppressed the EMT process, a key feature of cancer progression (54), which will be further analyzed below. The above is briefly summarized in **Table 1**.

**Table 1:** Summarizes the impact of IL-37 on cancer cell proliferation pathways.

First author/reference	Cancer type	Cell line(s)	Effect(s) associated with IL-37
Zhang Z et al. (2019)/ (48)	Hepatocellular carcinoma	SMMC7721 and Bel7402	Mitigation of STAT3 phosphorylation and suppression of STAT3 activity
Wang S et al. (2015)/ (47)	Cervical cancer	HeLa and C33A	Downregulation of STAT3 and pSTAT3
Yan X et al. (2017)/ (50)	Colorectal cancer	DLD1 and HT-29	Downregulation of β-catenin
Jiang Y et al. (2015)/ (46)	Renal cell carcinoma	A498 and Caki-1	Downregulation of IL-6 and pSTAT3 Y705
Liu R et al. (2016)/ (49)	Hepatocellular carcinoma	SMMC-7721, HepG2, Huh7, and MHCC-97H	Induction of G2/M cell cycle arrest; promotion of pSmad3C/p21 tumor-suppressive signaling
Mu X et al. (2021)/ (51)	Non-small cell lung cancer	A549	Regulation of m6A RNA methylation; inhibition of Wnt5a/5b pathway
Chen et al. (2016)/ (53)	Non-small cell lung cancer	A549	IL-37 reduces A549 cell proliferation, especially when combined with CCL22

### 3.2 The impact of IL-37 on apoptotic pathways in cancer

IL-37 has emerged as a crucial regulator of inflammatory responses (55). Recent studies have highlighted its potential influence on apoptotic pathways in cancer, primarily based on *in vitro* cell-line systems and xenograft models, where it may contribute to the regulation of cell death and survival (46, 56–58). Understanding the role of IL-37 in these processes could open new avenues for targeted therapies in oncology.

Jiang et al. investigated the role of IL-37 in RCC, highlighting its pro-apoptotic activity (46). Bcl-2, a proto-oncogene, plays a vital role in preventing apoptosis, thereby promoting cell survival (59). In RCC, Bcl-2 is often overexpressed, contributing to the resistance of cancer cells to conventional therapies such as chemotherapy and radiotherapy (59). Serum IL-37 levels are significantly lower in RCC patients and inversely correlated with tumor size and stage (46). *In vitro*, treatment of human RCC cell lines (A498 and Caki-1) with recombinant IL-37 induced apoptosis in a dose-dependent manner, as confirmed by Annexin V/7-AAD staining and flow cytometry. This apoptotic effect was accompanied by downregulation of anti-apoptotic and proliferative markers, including Bcl-2 and cyclin D1, and suppression of IL-6/STAT3 signaling. Overexpression of IL-6 reversed IL-37-induced apoptosis, reinforcing the central role of IL-6/STAT3 in IL-37-mediated tumor suppression. *In vivo*, IL-37 treatment reduced tumor growth and lowered IL-6 and HIF-1α expression, further supporting its antitumor and pro-apoptotic function (46). These findings suggest that IL-37 promotes apoptosis in RCC models and may have immunomodulatory potential, although its therapeutic role remains to be established in clinical studies. Li et al. revealed the significant role of IL-37 in regulating autophagy within HCC cells by modulating the PI3K/AKT/mTOR signaling pathway (57). IL-37 effectively inhibited HCC cell proliferation and promoted autophagy and apoptosis in the SMMC-7721 and Huh-7 cell lines. This regulatory effect is associated with reduction in the levels of phosphorylated proteins critical to the PI3K/AKT/mTOR pathway, including phosphorylated AKT (p-AKT), phosphorylated mTOR (p-mTOR), phosphorylated p70 ribosomal protein S6 kinase (p-p70S6K), and phosphorylated 4E-binding protein 1 (4E-BP1). The study demonstrated that IL-37 disrupted this crucial for cell growth and survival signaling axis, and that its impact on autophagy can be countered by the insulin-like growth factor 1 (IGF-1), an AKT agonist, or mimicked by a PI3K/AKT inhibitor (LY294002) (57). These findings highlight a potential role of IL-37 in modulating autophagy and apoptosis in HCC models by targeting autophagic and apoptotic pathways through a specific signaling mechanism.

Ouyang et al. investigated the role of IL-37 in promoting apoptosis in CC cells and its interaction with the pro-apoptotic factor Bim (58). They used HeLa and C33A CC cell lines to assess the effects of IL-37 overexpression on cell apoptosis and Bim expression. They revealed that transfection with the plasmid pIRES2-EGFP-IL-37 led to a significant increase in both IL-37 mRNA and protein levels in the HeLa and C33A cells. IL-37 treatment substantially increased the rate of apoptosis in both cell lines, with HeLa cells showing an approximate 153% increase and C33A cells a 25.4% increase in apoptosis rates. They also examined the effects of IL-37 on apoptosis-related genes. It was found that IL-37 upregulated the mRNA levels of Bim and Bax, both of which are pro-apoptotic factors, while it did not significantly affect the anti-apoptotic gene Bcl-2. Among these genes, Bim showed the most pronounced increase in expression. Further experiments using siRNA to silence Bim (SiBim) demonstrated that inhibition of Bim reduced its protein levels and decreased the apoptosis rates induced by IL-37. This suggested that Bim is a crucial mediator in the apoptosis process triggered by IL-37 (58). Overall, the study concludes that IL-37 promoted apoptosis in CC cells through the upregulation of Bim. These findings underscore the potential of IL-37 as a therapeutic agent for cervical cancer by enhancing cancer cell apoptosis.

Ding et al. explored the potential of IL-37 as a radiosensitizer for prostate cancer (PC), focusing on its effects on DU145 and PC-3 cell lines (56). Although IL-37 alone does not significantly impact PC cell growth, its combination with radiation therapy (RT) enhances the efficacy of the latest. Specifically, IL-37 improves RT-induced inhibition of cell proliferation and increased apoptosis in these PC cells. Through a series of techniques, including clonogenic survival assays, cell proliferation assays, immunohistochemistry, TUNEL staining, and caspase-3 activity assays the authors showed that IL-37 combined with RT reduced cell proliferation and increased cell death compared to RT alone. This combined treatment also led to changes in the expression of key regulatory molecules. For instance, IL-37/RT increased the levels of pro-apoptotic molecules such as Fas and Bax, and upregulated p27, while downregulating pro-proliferative molecules like cdk2 (56). These findings suggest that IL-37, when used alongside RT, can enhance the sensitivity of prostate cancer cells to radiation, potentially allowing for lower doses to be used while still achieving effective treatment. These indications of IL-37 as a potential radiosensitizer require further research to validate its clinical application.

In summary, IL-37 promotes apoptosis in cancer by downregulating survival proteins like Bcl-2 and Cyclin D1, disrupting the PI3K/AKT/mTOR pathway, upregulating pro-apoptotic factors like Bim, and enhancing the effectiveness of radiation therapy, suggesting it may represent a candidate for further therapeutic investigation. The above is briefly summarized in **Table 2**.

**Table 2:** Summarizes the impact of IL-37 on cancer-related apoptotic pathways.

Author/references	Cancer type	Cell line(s)	Effect(s) associated with IL-37
Jiang Y et al. (2015)/ (46)	Renal cell carcinoma	A498 and Caki-1	Downregulation of Bcl-2, and cyclin D1.
Ouyang P et al. (2019)/ (58)	Cervical cancer	HeLa and C33A	Upregulation of Bim
Ding VA et al. (2017)/ (56)	Prostate cancer	DU145 and PC-3	Upregulation of p27, Fas, and Bax, downregulation of cdk2
Li TT et al. (2017)/ (57)	Hepatocellular carcinoma	SMMC-7721 and Huh-7	Downregulation of p-AKT, p-mTOR, p-p70S6K, p-4E-BP1; regulation of autophagy via the PI3K/AKT/mTOR signaling pathway

### 3.3 IL-37’s role in cancer-related angiogenesis: a potential therapeutic target

Evidence linking IL-37 to angiogenesis arises from both human observational studies (e.g., serum or tissue correlations with VEGF and microvessel density) and preclinical models demonstrating direct effects on endothelial cells and tumor vascularization (11, 60). Recent studies reveal that IL-37 can effectively suppress angiogenesis by decreasing microvessel density (MVD) and vascular endothelial growth factor (VEGF) levels, highlighting its potential relevance as a therapeutic target, although current evidence is primarily derived from preclinical and correlative studies (61–64).

In their study, Li et al. investigated the involvement of IL-37 in multiple myeloma (MM) and its relationship with angiogenesis (63). They measured serum IL-37 levels in 45 newly diagnosed MM patients and 30 healthy controls. They found that serum IL-37 levels were significantly lower in MM patients compared to controls. This decrease in IL-37 was inversely related to the levels of angiogenesis markers such as VEGF and angiopoietin-2 (Ang-2), which were higher in MM patients and positively correlated with disease stage as classified by the International Staging System (ISS). They also found that IL-37 levels negatively correlated with both VEGF and Ang-2, suggesting that lower IL-37 levels are associated with increased angiogenesis in MM. Additionally, recombinant human (rh)IL-37 pretreatment of human umbilical vein endothelial cells (HUVECs) significantly reduced their tube formation capacity and decreased VEGF levels in the cell culture supernatant, demonstrating that IL-37 can inhibit angiogenesis *in vitro* (63). These findings suggest that IL-37 has a protective role against angiogenesis in MM. Lower serum levels of IL-37 are linked to higher angiogenesis markers, which may contribute to disease progression. Thus, IL-37 may have potential as a biomarker for assessing MM progression and a target for therapeutic strategies aimed at modulating angiogenesis in MM. Additionally, Li et al. investigated the role of intracellular mature IL-37 in tumor metastasis, with a focus on its effects on Rac1 activation and angiogenesis (62). They found that decreased IL-37 expression in lung adenocarcinoma tissues correlated with increased tumor metastasis and poorer patient prognosis. By analyzing clinical data from 84 patients, they established that lower IL-37 levels were significantly associated with higher metastatic potential and advanced tumor stages. *In vitro* experiments showed that overexpression of IL-37 inhibited the migration of BEL-7402 and HepG2 cells, while its knockdown enhanced cell migration, indicating that IL-37 plays a crucial role in suppressing tumor cell movement. The mechanism underlying IL-37’s anti-metastatic effects was explored further (62, 65). The study identified that the intracellular mature form of IL-37 (amino acids 46–218) is essential for its function. This form of IL-37 suppresses tumor cell migration by directly interacting with Rac1, a Rho GTPase that regulates the cytoskeleton and cell motility. Specifically, IL-37 binds to the CAAX motif in the C-terminal hypervariable region of Rac1, preventing its activation and membrane translocation. The inhibition of Rac1 signaling by IL-37 disrupts downstream pathways involved in cell movement and metastasis, effectively reducing tumor spread (65). *In vivo* experiments reinforced these findings by demonstrating that IL-37 overexpression significantly decreased tumor growth and metastasis in nude mice models. The reduction in metastatic spread was associated with lower Rac1 activation and decreased angiogenesis in the tumors. These results highlight the potential of intracellular mature IL-37 as a therapeutic agent for controlling Rac1 activity and limiting tumor progression (62). The study concludes that targeting the Rac1 signaling pathway through IL-37 could suggest a potential strategy for further investigation in the context of cancer metastasis.

Ge et al. investigated the role of IL-37 in non-small cell lung cancer (NSCLC), focusing on its impact on tumor growth and angiogenesis (61). IL-37 was examined as for its expression in NSCLC tissues compared to adjacent normal tissues. Using techniques real-time PCR, western blotting, and immunohistochemical staining, they found that IL-37 levels were significantly lower in NSCLC tissues. Reduced IL-37 expression was associated with advanced tumor stages, poorer prognosis, and was identified as an independent prognostic factor for overall survival in NSCLC patients. Further experimental work involved creating H1299 lung cancer cell lines with overexpressed IL-37 to study its effects on tumor progression. Although IL-37 did not directly affect cell proliferation or apoptosis *in vitro*, it significantly inhibited tumor growth in a xenografted mouse model. The study also revealed that IL-37 overexpression did not alter immune cell infiltration but resulted in reduced microvessel density and lower VEGF levels within the tumors. In addition, IL-37 directly inhibited the growth and capillary formation of human umbilical vein endothelial cells (HUVECs), suggesting an anti-angiogenic effect. The findings of this study highlight IL-37’s potential as a protective factor against lung cancer development through its role in inhibiting tumor angiogenesis (61). The data supports the hypothesis that IL-37’s anti-tumor effects are mediated by suppressing the formation of new blood vessels in the tumor microenvironment, making it a potential target for therapeutic strategies in NSCLC.

Finally, Mei et al. investigated the dual role of IL-37 in angiogenesis by examining its effects on different cell lines (64). They focused on HUVECs to evaluate IL-37’s impact on endothelial cell behavior. Recombinant IL-37 was found to promote angiogenesis in HUVECs, enhancing their migration and tubule formation, although these effects were slightly less pronounced than those induced by VEGF. Importantly, IL-37 did not alter HUVEC proliferation or apoptosis but activated the IL-1R8 signaling pathway, suggesting a specific role in endothelial cell function. In contrast, the study also examined IL-37’s effects on tumor-derived cells. IL-37-producing murine HCC cells were used to assess how IL-37 impacts angiogenesis in the TME. The presence of IL-37 in these tumor cells led to increased apoptosis of HUVECs and inhibited their migration and tubule formation. Supernatants from IL-37-producing HCC cells significantly impaired angiogenesis compared to those from control cells. *In vivo* experiments with a murine HCC model demonstrated that tumors overexpressing IL-37 had reduced vascular density and lower vascular permeability, indicating IL-37’s role in suppressing tumor angiogenesis. The study also revealed that IL-37 modulated the expression of angiogenic factors in tumor cells. Using microarray and qPCR analyses, they found that IL-37 down-regulated several proangiogenic factors, such as CYP1B1, FN1, MMP2, and PIK3CG, while up-regulating the antiangiogenic factor ANGPT1 in HCC cells. These changes in gene expression contribute to a shift from a proangiogenic to an antiangiogenic profile in tumor cells, thereby inhibiting overall tumor angiogenesis (64). This dual effect suggests that IL-37 can promote endothelial cell function directly but may act as an antiangiogenic agent within the tumor microenvironment, offering potential therapeutic insights for targeting angiogenesis in cancer. The above is briefly summarized in **Table 3**.

**Table 3:** Summarizes the effects of IL-37 on cancer-related angiogenesis.

First author/reference	Cancer type	Material studied	Effect(s) associated with IL-37
Mei et al. (2020)/ (64)	Hepatocellular carcinoma	SK-Hep-1, Hepa1-6, and SMMC-7721 cell lines	Downregulation of cytochrome P450 1B1 (CYP1B1), fibronectin 1 (FN1), matrix metalloproteinases MMP2 and MMP9, melanoma cell adhesion molecule (MCAM), phosphatidylinositol 3-kinase catalytic subunit gamma (PIK3CG), and vascular endothelial growth factor (VEGF); upregulation of angiopoietin 1 (ANGPT1); mitigation of M2 polarization of tumor-associated macrophages (TAMs)
Li ZC, et al. (2016)/ (63)	Multiple myeloma	Sera from 45 patients and 30 healthy controls	Negative correlation of IL-37 with angiopoietin 2 (Ang-2) and VEGF
Li et al. (2017)/ (62)	HCC, Acute monocytic leukemia	HepG2, BEL-7402, THP-1	Blocks Rac1: IL-37 binds Rac1, preventing its activation; Reduces Angiogenesis; Lowers Metastasis
Ge et al. (2016)/ (61)	NSCLC	H1299 cells	Reduces Microvessel Density; Inhibits VEGF: Lowers vascular endothelial growth factor levels; Blocks capillary structure formation in endothelial cells (HUVEC)

### 3.4 Impact of IL-37 on cancer cell migration, invasion, and EMT

IL-37 has emerged as a significant anti-inflammatory cytokine with profound implications in cancer biology (66). Recent studies have highlighted its role in modulating critical processes such as cell migration, invasion, and EMT all of which are pivotal in cancer progression and metastasis (53, 54, 67, 68). Exploring the influence of IL-37 on these processes could lead to the development of novel therapeutic strategies to limit cancer progression and enhance patient outcomes.

Chen et al. explored the impact of CCL22 and IL-37 on EMT in NSCLC A549 cells (53). EMT was assessed through changes in cell morphology and expression of key markers. Treatment with either CCL22 or IL-37 alone resulted in decreased expression of mesenchymal markers (vimentin and N-cadherin) and an increase in the epithelial marker E-cadherin, indicating a partial reversal of EMT. Microscopic observations supported these findings, showing that treated cells retained a cobblestone-like epithelial appearance with tighter intercellular junctions. The combination of both cytokines produced a more pronounced effect, with stronger suppression of mesenchymal traits and further upregulation of E-cadherin (53). These results suggest that CCL22 and IL-37, especially in synergy, effectively inhibit EMT in A549 cells and may be relevant in limiting tumor invasiveness, although this remains to be validated *in vivo* and clinically. Wang et al. investigated the role of IL-37 in endometrioid adenocarcinoma, focusing on its expression and effects on cell behavior (69). Initial findings showed that IL-37 mRNA and protein levels were upregulated in endometrioid adenocarcinoma tissues compared to normal endometrial tissues. However, immunohistochemistry (IHC) revealed that IL-37 protein expression was significantly downregulated in adenocarcinoma cells. Further analysis indicated that IL-37 expression was related to factors like age and myometrial invasion, but not to tumor differentiation or stage. They explored the impact of IL-37b on endometrial cancer cell lines. Overexpression of IL-37b in Ishikawa cells, which naturally had low IL-37 levels, did not affect cell proliferation or colony formation. However, it significantly suppressed the migration and invasion of these cells. Conversely, knocking down IL-37 in AN3CA cells, which had higher baseline levels of IL-37, increased their migratory and invasive abilities. This suppression of migration and invasion by IL-37b was linked to a reduction in matrix metalloproteinase 2 (MMP2) expression through the Rac1/NF-κB signaling pathway (69). *In vivo* experiments further supported these findings. A peritoneal metastatic xenograft model in mice demonstrated that stable expression of IL-37b in Ishikawa cells led to a significant reduction in tumor metastasis, particularly in the liver, without affecting the overall health or weight of the mice (69). They conclude that IL-37b plays a critical role in inhibiting the migration, invasion, and metastasis of endometrial cancer cells, making it a potential therapeutic target for treating endometrioid adenocarcinoma. Additionally, Wu et al. investigated the role of IL-37 in gallbladder cancer (GBC) by analyzing its expression and effects on cancer cell behavior (70). They found that IL-37 levels were significantly lower in GBC cell lines (GBC-SD and NOZ) compared to a non-cancerous biliary epithelial cell line (H69). Both mRNA and protein levels of IL-37 were reduced in the cancerous cells, indicating a potential role for IL-37 in the progression of GBC. To assess the impact of IL-37 on cancer cell migration and invasion, they involved overexpressing IL-37 in GBC-SD and NOZ cells. Results from transwell and invasion assays demonstrated that IL-37 overexpression led to a notable reduction in the migration and invasion of these cells. This suppression was dose-dependent, suggesting that higher levels of IL-37 could more effectively inhibit these cancer processes. Further analysis revealed that IL-37 affects EMT by altering the expression of key EMT markers. Overexpression of IL-37 increased levels of E-cadherin, an epithelial marker, while decreasing levels of snail and vimentin, associated with mesenchymal cells. Additionally, IL-37 was found to suppress HIF-1α, a regulator of EMT (71). The study also demonstrated that stabilizing HIF-1α with CoCl2 diminished the inhibitory effects of IL-37 on cell migration and invasion, highlighting that IL-37’s anti-cancer effects are likely mediated through HIF-1α inhibition (70). These findings suggest that IL-37 could be a possible therapeutic target for mitigating GBC metastasis.

Ouyang et al. explored the role of IL-37 in inhibiting the invasion of cervical cancer (CC) cells and its relationship with the runt-related transcription factor 2 (RUNX2) (72). They found that IL-37 significantly reduced the mRNA and protein expression of RUNX2, a molecule associated with increased tumor invasion (73). IL-37 was shown to decrease RUNX2 levels by 78.5% in SiHa cells and 61.5% in C33A cells. This reduction in RUNX2 corresponded with a notable decrease in the invasive potential of these cancer cells, with a 36.23% and 26.21% reduction in invasion observed in SiHa and C33A cells, respectively. Further analysis indicated that the overexpression of RUNX2 partially counteracted the inhibitory effects of IL-37 on cell invasion. In the presence of high RUNX2 levels, the reduction in invasion caused by IL-37 was reversed to 86.62% in SiHa cells and 87.08% in C33A cells. This suggests that RUNX2 plays a significant role in mediating the effects of IL-37 on cancer cell invasion (72). Overall, the study highlights the potential of IL-37 as an anti-cancer agent, particularly in relation to its ability to downregulate RUNX2 and inhibit cervical cancer cell invasion. These findings contribute to a better understanding of IL-37’s role in cancer progression and suggest that targeting RUNX2 could be a viable strategy for enhancing the therapeutic effects of IL-37 in cervical cancer. Finally, Jiang et al. investigated the effects of IL-37 on the invasion and metastasis of NSCLC by targeting the IL-6/STAT3 signaling pathway (74). They found that IL-37 levels were significantly lower in the plasma of NSCLC patients compared to healthy controls, and this decrease correlated with advanced tumor stages. *In vitro* experiments using the A549 NSCLC cell line showed that IL-37 inhibits both invasion and migration, while IL-6 promotes these processes. IL-37’s inhibition of cancer progression was linked to its suppression of STAT3 activation, a key factor in promoting EMT (75), which is crucial for cancer metastasis. Specifically, the study demonstrated that IL-37 reduced the expression of pSTAT3, vimentin, and N-cadherin—markers associated with EMT—while increasing E-cadherin levels, which are indicative of a more epithelial and less invasive state. These effects were reversed when IL-6 was added, underscoring IL-37’s role in counteracting IL-6-driven tumor progression (74). The findings suggest that IL-37 functions as a tumor suppressor in NSCLC by modulating the IL-6/STAT3 signaling pathway, suggesting it may be a candidate for further investigation in the context of NSCLC invasion and metastasis. Collectively, these findings are largely derived from preclinical models, with limited validation in human cohorts. The above is briefly summarized in **Table 4**.

**Table 4:** Summarizes the effects of IL-37 in cancer-related cell migration, invasion and EMT.

First author/reference	Cancer type	Cell line(s)	Effect(s) associated with IL-37
Wang X et al. (2021)/ (69)	Endometrial adenocarcinoma	Ishikawa, HEC-1-A, AN3CA, and RL95-2	Downregulation of MMP2 via the Rac1/NF-κB pathway
Wu TJ et al. (2018)/ (70)	Gallbladder cancer	GBC-SD and NOZ	Downregulation of vimentin and Snail, upregulation of E-cadherin via attenuation of HIF1α
Jiang et al. (2018)/ (74)	Non-small cell lung cancer (NSCLC)	A549	Inhibits IL-6 Production; Suppresses STAT3 Activation; Reduces EMT Markers (vimentin, N-cadherin); Increases E-cadherin Expression; Impedes Cell Migration and Invasion
Ouyang P et al. (2019)/ (72)	Cervical cancer	Siha and C33A	Downregulation of RUNX2
Chen Y et al. (2016)/ (53)	Non-small cell lung cancer (NSCLC)	A549	Decreased vimentin and N-cadherin; increased E-cadherin; inhibition of EMT; preservation of epithelial morphology

## 4 IL-37 and innate immunity: exploring cellular interactions in cancer

The effects of IL-37 on innate immune cells in cancer have been predominantly characterized in experimental systems, with human evidence remaining limited and largely associative.

### 4.1 Natural killer cells

IL-37 plays a significant role in modulating also innate immunity within local TME. Landolina et al. investigated the role of IL-37 in modulating the function of NK cells, key players in anti-tumor immunity (76). Traditionally recognized for its anti-inflammatory properties, IL-37 demonstrated unexpected effects on NK cell activity. A primary finding was the ability of IL-37 to enhance NK cell cytotoxicity against various tumor cell lines including the human colon cancer cell lines HT-29, SW480, and COLO-205, the human neuroblastoma cell line IMR-32 and K562, a human chronic myelogenous leukemia cell line. This increased killing capacity was linked to a reduction in the expression of IL-1R8, a receptor known to inhibit NK cell function. By downregulating IL-1R8, IL-37 effectively removed a brake on NK cell activity, allowing for a more robust anti-tumor response. They further elucidated the mechanism underlying this enhancement. IL-37, in combination with the activating cytokine IL-15, stimulated key signaling pathways within NK cells, including ERK and NF-κB. These activated pathways contributed to the observed increase in NK cell cytotoxicity and cytokine production. Additionally, they correlated higher IL-37 expression levels with improved survival rates in patients with colon adenocarcinoma and neuroblastoma. This suggests that IL-37 might serve as a prognostic biomarker and a potential therapeutic target in these cancers. In conclusion, the study provided novel insights into the complex role of IL-37 in regulating NK cell function (76). By unveiling the mechanisms through which IL-37 enhanced NK cell anti-tumor activity, the research offers promising avenues for developing novel immunotherapies for cancer. Zhao et al. explored the role of IL-37 in HCC with a particular focus on its effects on NK cells (77). It was observed that high levels of IL-37 expression in tumor tissues were associated with an increased density of CD57+ NK cells. This correlation was specific to NK cells and did not extend to CD3+ or CD8+ T cells, highlighting IL-37’s selective influence on NK cell recruitment. *In vitro* experiments supported these findings, showing that supernatants from HCC cells overexpressing IL-37 attracted significantly more NK cells compared to control supernatants. This suggested that IL-37 enhanced the ability of HCC cells to recruit NK cells. *In vivo* studies using mouse models further confirmed IL-37’s role in promoting NK cell activity. Mice injected with HCC cells engineered to overexpress IL-37 exhibited reduced tumor growth and a greater infiltration of CD57+ NK cells into tumor tissues. This indicated that IL-37 not only attracted NK cells but also enhanced their antitumor effects. Furthermore, NK cell depletion experiments revealed that the antitumor benefits of IL-37 were significantly reduced when NK cells were absent. Mice lacking NK cells showed accelerated tumor growth and increased tumor weight, underscoring the critical role of NK cells in mediating IL-37’s antitumor activity (77). Overall, the study demonstrated that IL-37 plays a crucial role in modulating the tumor microenvironment by recruiting and activating NK cells, suggesting its potential utility in immunotherapy for HCC.

### 4.2 Macrophages

IL-37 possesses significant effects also on macrophage function (78–80). IL-37 impairs host resistance to Listeria monocytogenes by reducing macrophage bactericidal activity and increasing apoptosis (78). It also modulates liver inflammation and fibrosis by downregulating hepatic Kupffer and stellate cell activation, which may help prevent liver cancer (79). Furthermore, in atherosclerosis, macrophage-specific IL-37 expression decreases inflammation and plaque formation (80). IL-37 reduces inflammation in macrophages by inhibiting the NF-κB pathway and decreasing pro-inflammatory cytokines IL-1β, TNF-α, and IL-6 (81). It interferes with the MyD88-TRAF6 signaling pathway, reducing TRAF6 ubiquitination and NF-κB activation (81). IL-37’s expression is increased by dexamethasone, further aiding its anti-inflammatory effects. Understanding IL-37’s role in these inflammatory processes offers valuable insights into its potential therapeutic applications in chronic inflammatory diseases and cancer.

Zhang et al. investigated the role of IL-37 in HCC, focusing on its impact on the polarization of tumor-associated macrophages (TAMs) (82). HCC patient-derived peripheral blood mononuclear cells (PBMCs) exhibited decreased IL-37 expression, low numbers of M1, and high numbers of M2 macrophages. To elucidate IL-37’s role in macrophage polarization, TAMs from HCC patients were transfected to either overexpress or knock down IL-37. Overexpression of IL-37 increased M1 (iNOS and CD86) and decreased M2 (ARG1 and CD206) markers, while IL-37 knockdown produced the opposite effect. Further, HCC cell lines HepG2 and Huh-7 cultured with conditioned media from these modified TAMs showed that IL-37 overexpression inhibited cell proliferation, migration, and invasion, whereas IL-37 knockdown enhanced these processes. They also explored the IL-6/STAT3 pathway, finding that recombinant human IL-6 (rhIL-6) treatment counteracted the effects of IL-37 overexpression, promoting M2 polarization and tumor cell growth. In a mouse xenograft model, IL-37 overexpression in TAMs led to reduced tumor growth, with increased M1 and decreased M2 markers in tumors (82). These results suggest that IL-37 suppressed HCC growth by inhibiting M2 polarization of TAMs via the IL-6/STAT3 signaling pathway, unveiling a potential therapeutic target for HCC.

### 4.3 Neutrophils

Currently, there is a limited body of research evidence suggesting that IL-37 plays a role in regulating neutrophils and influencing cancer progression. For example, Zhu et al. found that low IL-37 expression and high levels of CD66b+ neutrophils are associated with poorer survival outcomes in CRC patients (83).

Li et al. demonstrated that IL-37 plays a critical role in mitigating the pro-tumor effects of neutrophils influenced by circulating tumor cells (CTCs) (84). In the context of cancer progression, CTCs can trigger systemic inflammation, leading to the activation of neutrophils in a way that promotes tumor growth and metastasis (85). They documented that IL-37 counteracted this by reducing the production of inflammatory cytokines like IL-6 and G-CSF, which are essential for converting neutrophils from a tumor-suppressing to a tumor-promoting state. IL-37 prevented the upregulation of genes associated with tumor promotion in neutrophils, such as Mmp9, Bv8, Arg1 and Nos2, and preserved their tumor-suppressive function. Additionally, IL-37 inhibited the activation of inflammatory pathways driven by CTCs by the secretion of IL-6 and G-CSF, reducing the overall systemic inflammation and neutrophil infiltration in tumor sites (84). By curbing these pro-tumor mechanisms, IL-37 helped maintain the anti-tumor potential of neutrophils, making it a promising therapeutic target for controlling cancer metastasis.

A recent study of Guo et al. elucidated the role of IL-37 in modifying neutrophil behavior and its broader implications for cancer progression (86). They investigated the role of IL-37d in regulating C/EBPb (CCAAT/enhancer-binding protein beta) and its impact on tumor progression and survival in a murine model of lung cancer. The key finding was that IL-37d directly binds to the DNA-binding domain (DBD) of C/EBPb, rather than interacting with COP1 (constitutive photomorphogenic 1), which is known for promoting C/EBPb degradation (87). IL-37d binding to C/EBPb impaired its ability to bind DNA, thereby inhibiting its transcriptional activity. The data showed that IL-37d did not interact directly with COP1 but modulated the C/EBPb-COP1 interactions by affecting C/EBPb’s DNA binding ability. They further elucidated the mechanism by which IL-37d enhanced C/EBPb ubiquitination and degradation through its interaction with COP1. The binding of IL-37d to C/EBPb was found to prevent C/EBPb from binding to DNA probes and reduced its nuclear levels, indicating a significant reduction in C/EBPb’s transcriptional activity. These findings demonstrate that IL-37d disrupts C/EBPb’s function by targeting its DBD, thereby promoting its degradation. In terms of tumor progression, IL-37d treatment notably inhibited the migration of both tumor cells and neutrophils. In a murine model with LLC (Lewis lung carcinoma) cells, IL-37d administration led to a significant reduction in tumor cell migration and extended the survival of tumor-bearing (TB) mice. A medium dose of IL-37d was particularly effective in prolonging survival and reducing tumor burden. The study also observed that IL-37d reduced neutrophil infiltration into the lungs at early stages of tumor growth, highlighting its role in suppressing spontaneous neutrophil migration and consequently slowing tumor progression. Moreover, IL-37d treatment resulted in decreased levels of C/EBPb and its downstream target S100A9 in the lungs of 1- and 2-week TB mice. However, this effect diminished by the third week, suggesting that IL-37d’s influence on C/EBPb levels may vary with tumor progression stages (86). In conclusion, IL-37d directly binds to the DBD of C/EBPb, disrupting its DNA binding and transcriptional activity. By promoting C/EBPb degradation through COP1 and inhibiting neutrophil migration, IL-37d effectively reduces tumor cell migration and extends the survival of tumor-bearing mice (86). This study highlights IL-37d’s potential as a therapeutic agent in cancer treatment by modulating both immune responses and tumor progression.

### 4.4 Dendritic cells

IL-37 exerts context-dependent effects on dendritic cells, which can translate into either suppression or enhancement of anti-tumor immunity depending on the DC subset and tissue microenvironment. In line with its well-known anti-inflammatory properties, IL-37 can restrain DC activation and promote tolerogenic functions, but, under certain conditions, it can also support DC-mediated priming of cytotoxic T cells and anti-tumor responses. Research has demonstrated that IL-37 plays a crucial role in controlling dendritic cells, leading to significant modulation of adaptive immune responses (88). In IL-37tg mice, DCs exhibit reduced expression of key molecules necessary for T cell activation and produce fewer pro-inflammatory cytokines. Instead, these DCs promote the induction of Treg cells, resulting in a dampened immune response (88) and highlighting IL-37’s potential in managing inflammatory disease and cancer. Additionally, The IL-37 inhibits dendritic cell maturation via the IL-1R8-TLR4-NF-κB pathway, reducing atherosclerosis in ApoE−/− mice (89). This suggests that targeting this pathway could be a potential therapeutic strategy for atherosclerosis.

In the context of cancer, IL-37 significantly influences dendritic cells in two key ways (90, 91). Liu et al. investigated the role of IL-37 in enhancing anti-tumor immunity through its impact on DCs and cytotoxic T lymphocytes (CTLs) in HCC. IL-37 was found to significantly influence the immune response against HCC by modulating DC function and enhancing CTL activity. IL-37 indirectly boosted the cytotoxicity of CTLs by acting on DCs, which are critical for antigen presentation and T cell activation. In co-culture experiments, DCs treated with the supernatant from Hep3B cells overexpressing IL-37 demonstrated a notable increase in IFN-γ secretion by T lymphocytes compared to those treated with controls. This enhancement in IFN-γ production was associated with a higher proportion of IFN-γ+ T cells and IFN-γ+ CD8+ T cells, leading to increased CTL-mediated cytotoxicity against HCC cells. Additionally, IL-37 promoted the maturation of DCs, as evidenced by increased expression of major histocompatibility class I and II (MHC I and II), CD80, CD86, and CD40 on these cells. This indicates a more effective antigen-presenting capability. Additionally, IL-37-treated DCs showed elevated levels of chemokines CXCL9 and CXCL10, which are essential for recruiting T cells to the tumor site. IL-37 also upregulated the secretion of key cytokines such as IL-2, IL-12, IL-12p70, IFN-α, and IFN-γ, further supporting a robust anti-tumor immune response. *In vivo* studies using Hepa 1–6 cells overexpressing IL-37 revealed that IL-37 enhanced DC recruitment to the TME and inhibited tumor growth. Mice injected with Hepa 1–6 cells overexpressing IL-37 exhibited slower tumor growth, reduced tumor volume and weight, and significantly higher infiltration of CD11c+ DCs compared to controls. However, there was no significant difference in the numbers of intratumoral CD4+ and CD8+ T cells (90). In summary, IL-37 enhanced the anti-tumor immune response in HCC by improving DC maturation and function, increasing cytokine secretion, and indirectly boosting CTL cytotoxicity. These findings underscore IL-37’s potential as a therapeutic agent in cancer immunotherapy.

Zeng et al. explored the role of IL-37 in skin squamous cell carcinoma (SCC) induced by DMBA/TPA, focusing on its impact on DCs and CD8+ T cells where they identified a significant positive correlation between CD8+ T cells and CD103+ DCs in the DMBA/TPA-induced SCC model (91). Specifically, a signature of CD103+ DCs, which includes markers such as IRF8, FLT3L, and CLEC9A, was positively associated with the levels of CD8A and chemokines CXCL9 and CXCL10. This correlation underscores the critical role of CD103+ DCs in activating CD8+ T cells and supporting anti-tumor immunity. They further demonstrated that IL-37 exerted a negative impact on CD103+ DCs by inhibiting their glycolysis through the SIGIRR-mediated signaling pathway. This inhibition led to reduced DC maturation and migration, which in turn impaired their ability to activate CD8+ T cells effectively. In IL-37tg mice, CD103+ DCs showed decreased expression of CD40 and CCR7, lower secretion of CXCL9 and CXCL10, and diminished migration to skin-draining lymph nodes (SDLN). These findings suggest that in this distinct DC subset and tissue context, IL-37’s effects on DCs compromise their function, supporting a tumor-promoting role of IL-37 in this model (91). Mechanistic investigations revealed that IL-37 activated AMPK and inhibited the Akt/mTOR signaling pathway in CD103+ DCs, thereby reducing glycolysis. This metabolic alteration impaired the functional capabilities of DCs and affected their role in supporting CD8+ T cell responses. Consequently, IL-37tg mice exhibited increased tumor growth and a reduced number of CD103+ DCs, correlating with a less effective anti-tumor immune response and enhanced SCC development. The study’s results highlight a novel mechanism through which IL-37 promotes skin cancer by interfering with CD103+ DC metabolism. By targeting the SIGIRR-AMPK-Akt signaling pathway, IL-37 impaired DC function and CD8+ T cell activation, contributing to tumor progression (91). Taken together, these studies illustrate the dual, highly context-dependent nature of IL-37’s effects on DC biology and anti-tumor immunity which should be carefully considered when targeting IL-37 or its signaling pathways therapeutically.

## 5 The role of IL-37 in shaping the immune landscape of cancer

### 5.1 Investigating the interaction between IL-37 and T cells

Growing evidence suggests IL-37 influences T and NK cells in various inflammatory processes (92, 93), potentially promoting carcinogenesis. Its regulatory role in the immune system may contribute to cancer development and progression. Feng et al. investigated the role of IL-37 in T cell-dependent liver injury (92). They found that IL-37 effectively suppressed the persistent hepatic production of IFN-γ and TNF-α, which are crucial in causing hepatocyte apoptosis and liver fibrosis. IL-37 achieved this by inhibiting the IFN-γ/TLR4-induced M1 macrophage activation, leading to reduced levels of pro-inflammatory cytokines like TNF-α, IL-1β, and IL-12. Additionally, IL-37 enhanced the Th2 response, increasing IL-4 and IL-13 levels, which promote M2 macrophage activation. This further diminished pro-inflammatory cytokine expression and boosted anti-inflammatory cytokines IL-10 and IL-1Ra. Consequently, IL-37 reduced liver injury, inflammation, and fibrosis by modulating macrophage activity and cytokine expression, demonstrating its potential as a therapeutic agent in T cell-dependent liver diseases like autoimmune hepatitis (92). On the other hand, IL-37’s impact on colitis varied with environmental conditions (93). In conventionally housed mice, IL-37 exacerbated DSS-induced colitis by compromising the epithelial barrier, increasing cell apoptosis, and reducing antibacterial gene expression. This resulted in intensified inflammation with higher neutrophil and NK cell infiltration and increased proinflammatory cytokines like IL-17 and IFN-γ. Conversely, in specific pathogen-free (SPF) conditions, IL-37 appeared protective, suggesting its effects are influenced by the gut microbiome (93). Thus, IL-37’s role in colitis is shaped by both environmental factors and microbial community composition and, given the tight link between chronic colitis and colorectal carcinogenesis, these context-dependent effects may translate into either tumor-promoting or tumor-restraining outcomes in the intestine.

Additional evidence suggests that IL-37 may directly suppress cytotoxic CD8+ T-cell function. Liu et al. showed that exogenous IL-37 reduced HBV peptide–induced perforin and granzyme B secretion by CD8+ T cells from patients with acute hepatitis B and dampened their cytotoxic activity in a cell contact dependent manner, without significantly altering pro-inflammatory cytokine secretion or PD-1/CTLA-4 expression (PMID:36326182). This study supports the concept that IL-37 can directly attenuate CD8+ T-cell effector function. This observation is particularly important when considering the tumor microenvironment, where diminished cytotoxic T-cell activity may favor immune escape.

Additionally, increasing evidence suggests that IL-37, through its ability to modulate chronic inflammation and immune responses, could ultimately shape cancer initiation and progression. In oral squamous cell carcinoma (OSCC), high IL-18/IL-37 ratio in serum correlated with advanced tumor stages and poorer survival, suggesting IL-18 promotes tumor progression (94). Elevated IL-18 in serum was linked to increased CD19+ B cells, while reduced IL-37 was associated with fewer CD3+CD8+ T cells, indicating disrupted adaptive immune responses (94). Observations like these have prompted intense studies into the role of IL-37 in regulating T cells and NK cells, highlighting its potential impact on immune responses in cancer. In IL-37tg mice, as detailed in the study by Wang et al. (95), the colon epithelium maintained normal characteristics with stable cell proliferation, apoptosis rates, and crypt lengths, and there were no notable differences in goblet cell numbers or tight junction protein levels compared to wild-type (WT) mice. The mucin production and immune cell distribution remained comparable to WT, and inflammatory cytokine levels were similar under normal conditions. However, IL-37tg mice displayed increased susceptibility to CRC development in the AOM/DSS model, evidenced by a greater tumor burden, larger tumors, and more severe pathology. Histological analysis showed elevated tumor cell proliferation, heightened Stat3 signaling, and decreased apoptosis in IL-37tg mice, suggesting that IL-37 promoted tumor growth. While the number of CD8+ T cells in tissue was consistent between both groups, IL-37tg mice exhibited impaired CD8+ T cell activation and functionality, characterized by reduced proliferation (Ki-67), effector markers (CD44, Granzyme B, Perforin), and tumor-specific responses (IFN-γ, TCRs). IL-37 interfered with IL-18–induced CD8+ T cell functions through SIGIRR, contributing to a more favorable environment for tumor development (95). Thus, while IL-37 maintained epithelial stability, it nevertheless enhanced colitis-associated carcinogenesis by modulating immune responses. Osborne et al. investigated the expression and regulation of IL-37 in melanoma patients (96). They found that IL-37 mRNA levels were significantly elevated in the blood of melanoma patients compared to healthy individuals. Among immune cell subsets, Tregs exhibited the highest IL-37 expression, which was further increased in melanoma patients. Additionally, B cells and NK cells also showed elevated IL-37 levels in patient blood, while monocytes and granulocytes did not. Using *in vitro* experiments, the authors demonstrated that melanoma-conditioned media (MCM) induced IL-37 expression at both the mRNA and protein levels in several lymphocyte populations, with the most pronounced effect in Tregs. To determine the key mediators, they blocked interleukin-1 receptor (IL-1R) signaling in melanoma cells during MCM preparation, which significantly reduced IL-37 induction. Further analysis using a cytokine array and ELISA revealed that transforming growth factor-β (TGF-β) secretion was notably diminished under IL-1R blockade. Finally, neutralizing TGF-β with a specific antibody (1D11) also abrogated IL-37 upregulation in Tregs, confirming that tumor-derived TGF-β is a major driver of IL-37 expression (96). They concluded that melanoma-secreted factors, especially TGF-β, enhance IL-37 expression in immune cells—primarily in Tregs—providing another mechanism through which IL-37 drives tumor-promotion, specifically through immunosuppression. In a study led by Mei et al., IL-37 was found to significantly influence the TME by modulating the metabolic functions of myeloid-derived suppressor cells (MDSCs) (97). RNA sequencing (RNA-seq) of MDSCs treated with recombinant IL-37 (rIL-37) showed that IL-37 treatment was associated with upregulation of 129 and downregulation of 163 genes. Gene set enrichment analysis (GSEA) indicated that IL-37 treatment enriched pathways related to inflammation and receptor activity in MDSCs. Furthermore, IL-37 enhanced both glycolysis and oxidative phosphorylation (OXPHOS) in MDSCs, leading to increased ATP production and leakage. This extracellular ATP impaired the immunosuppressive function of MDSCs, resulting in elevated T cell proliferation and effector cytokine production. IL-37 treatment specifically led to several modifications in T cells, including enhanced effector function, increased cytotoxic capacity, reduced PD-1 expression, and improved activation, all indicative of a less exhausted T cell phenotype. The released ATP, due to IL-37-induced metabolic changes in MDSCs, further stimulated T cell proliferation and activation (97). Collectively, these results suggest that IL-37 enhanced anti-tumor T cell responses by reprogramming MDSC metabolism, thus reducing their immunosuppressive capabilities and fostering a more effective anti-tumor immune response contrasting its tumor-promoting effects in melanoma and CRC. Together the above studies highlight IL-37’s context-dependent dual role in cancer.

### 5.2 Exploring the interaction between IL-37 and B cells

The immunoregulatory role of IL-37 in B cells is essential for understanding its therapeutic potential in inflammation and autoimmune diseases. A study by Wang et al. demonstrated that human regulatory B cells (Bregs) suppress autoimmune diseases primarily through IL-37, independent of IL-10, via the hypoxia-inducible factor-1a (HIF-1a) pathway (98). In IL-37 transgenic mice, IL-37 reduced pro-inflammatory cytokines and suppressed T cell responses, while IL-37-producing Bregs enhanced immunoregulation. Specifically, CpG-induced IL-37 expression in CD19+ B cells was shown to modulate T cell proliferation, offering insights into immune homeostasis (98). These findings highlight IL-37’s potential in modulating B cell function underscoring further its relevance in translational research.

Aging is commonly associated with chronic, low-grade inflammation, which adversely affects various physiological processes, including B lymphopoiesis. This age-related inflammatory environment in the bone marrow (BM) is characterized by elevated levels of cytokines such as TNF-α and IL-6, contributing to impaired B cell progenitor function (99). This inflammatory milieu leads to a reduction in the metabolic and proliferative capacity of these progenitors, contributing to their impaired fitness and function (100). Specifically, aged B progenitors exhibit reduced expression of genes involved in nucleotide synthesis and cell-cycle regulation, such as Hprt and Gmps, and decreased IL-7R signaling, which is crucial for B cell lineage commitment.

Transgenic expression of human IL-37 in mice (IL-37tg, as described above) has shown protective effects against several inflammatory conditions, making it a promising candidate for mitigating age-associated inflammatory impacts on B cell development and oncogenesis (101). Studies have demonstrated that IL-37 has potent anti-inflammatory properties that can ameliorate the detrimental effects of aging-associated inflammation on B cell progenitors (101). In aged IL-37tg mice, the expression of IL-37 significantly reduced the levels of pro-inflammatory cytokines like TNF-α, IL-6, and IL-1β in the BM and serum. This reduction in inflammation helped preserve the functional capacity of B cell progenitors, as evidenced by maintained STAT5 activation and the expression of metabolic and cell-cycle genes comparable to those observed in young mice.

A key concern with aging and chronic inflammation is the increased risk of oncogenesis. The inflammatory environment in aged BM not only impairs B cell progenitor function but also creates selective pressures that favor the emergence and expansion of oncogenically transformed cells (101). For instance, in aged mice, the expression of oncogenic mutations such as NRASV12 was selectively favored in B progenitor cells within the inflammatory milieu. These oncogenically initiated cells exhibited enhanced proliferation and survival, contributing to the increased risk of hematologic malignancies in the elderly (101). IL-37 plays a crucial role in mitigating this risk. In aged IL-37tg mice, the reduction in inflammation prevented the selection and expansion of NRASV12 -expressing B progenitor cells. This indicates that IL-37 not only preserved the fitness of normal B cell progenitors but also reduced the oncogenic potential by maintaining a less inflammatory BM environment. The protective effect of IL-37 against oncogenesis was further evidenced by the maintenance of normal gene expression profiles and signaling pathways in B progenitors, which counteracted the selective advantages conferred by oncogenic mutations. In more detail, when young BM progenitor cells were transplanted into aged IL-37tg mice, these cells exhibit improved fitness parameters compared to those transplanted into non-transgenic aged mice. The presence of IL-37 prevented the aging-related decline in key gene expressions, such as Hprt, Gmps, and Stat5b, and maintained proper STAT5 signaling. Moreover, IL-37 expression in aged mice also prevented the selection of oncogenic NRASV12-expressing B progenitor cells, which typically occurred in an inflammatory aging context, suggesting that IL-37 helped mitigate the selective pressures that favor oncogenic transformations in B cells. In further support of IL-37’s role in oncogenesis prevention, IL-37 transgenic (IL-37tg) mice displayed a significant reduction in the expression of oncogenic markers and inflammatory mediators. Flow cytometric analyses revealed lower production of TNF-α in pro–B cell progenitors isolated from aged IL-37tg mice compared to non-transgenic aged controls. Additionally, sorted pro–B cell progenitors from these mice exhibited reduced mRNA expression of Ifnz and IFN-α–inducible protein 27 (Ifi27), both of which are associated with inflammation-driven oncogenesis. Further evidence comes from examining B cell development in TNF-αΔARE mice, which exhibit heightened constitutive TNF-α expression and increased susceptibility to chronic inflammatory diseases. Young TNF-αΔARE mice showed significantly impaired B lymphopoiesis and increased myeloid cell frequency, similar to aging phenotypes. This impairment was linked to reduced expression of Hprt and Gmps in pro–B cells, underscoring the impact of chronic inflammation on oncogenic transformation (101). In conclusion the interplay between IL-37 and B cell progenitors highlights its tumor-suppressive role here through attenuating chronic inflammation that regulates B lymphopoiesis and oncogenesis during aging. IL-37’s ability to preserve B cell progenitor fitness offers a promising therapeutic strategy to counteract the adverse effects of aging on the immune system (101).

## 6 Tumor-promoting and immunosuppressive functions of IL-37

Building on the context-dependent effects already described, accumulating evidence further reveals specific mechanisms through which IL-37 promotes tumor progression. Such effects are most evident in settings where IL-37 weakens immune surveillance mechanisms, particularly cytotoxic CD8^+^ T-cell activity and dendritic cell–mediated antigen presentation or shifts immune responses toward tolerance rather than tumor elimination.

A well-characterized example of this phenomenon is observed in inflammation-associated colorectal cancer. In the azoxymethane/dextran sodium sulfate (AOM/DSS) model of colitis-associated carcinogenesis, IL-37 transgenic mice developed increased tumor burden and more severe disease compared with wild-type controls (PMID: 35046386). Importantly, mechanistic analyses demonstrated that IL-37 was dispensable for intestinal mutagenesis and did not primarily promote tumorigenesis directly on epithelial cells. Instead, IL-37 impaired immune-mediated tumor control, mainly by reducing the functional capacity of CD8^+^ cytotoxic T cells. This impairment was characterized by diminished T-cell activation and reduced expression of effector molecules required for effective tumor cell killing. Mechanistically, IL-37 downregulated the IL-18–dependent CD8^+^ T-cell activation through a SIGIRR-mediated inhibitory pathway, leading to attenuated downstream signaling and weakened cytotoxic function(PMID: 35046386). Beyond its effects on cytotoxic T cells, IL-37 can reinforce immunosuppressive lymphocyte programs (PMID: 31099111). Experimental exposure of lymphocytes to melanoma-conditioned media increased IL-37 expression, particularly within the Treg compartment, a response that was at least partly driven by TGF-β–dependent signaling (PMID: 31099111). Importantly, this highlights the role of immune-cell–derived IL-37, rather than tumor-cell–derived IL-37 alone, in shaping a tolerance-promoting immune environment. DCs represent another critical pathway through which IL-37 may support tumor progression (PMID: 25294929). Experimental models demonstrate that IL-37 expression in DCs promotes a tolerogenic phenotype, characterized by reduced antigen presentation and weakened costimulatory signaling. IL-37–expressing DCs showed diminished upregulation of molecules such as MHC class II and CD40, produced lower levels of immunostimulatory cytokines (including IL-12), and secreted higher levels of IL-10. Functionally, these changes resulted in inefficient priming of antigen-specific T cells and enhanced induction of Tregs. *In vivo*, this DC phenotype was associated with reduced accumulation of CD8^+^ T cells and increased immune tolerance, providing a clear mechanism by which IL-37 may suppress effective anti-tumor immunity(PMID: 25294929). Consistent with this DC-centered mechanism, studies in chemical skin carcinogenesis using the DMBA/TPA model showed that IL-37 can accelerate tumor development by selectively impairing a key dendritic cell subset (PMID: 36891351). In IL-37 transgenic mice, tumor formation occured earlier and more extensively, accompanied by a reduction in CD103^+^ cross-presenting dendritic cells (cDC1), which are essential for effective CD8^+^ T-cell priming. Mechanistically, IL-37 signals through SIGIRR altered the metabolic state of these DCs by activating AMPK and inhibiting Akt/mTOR-driven glycolysis. This metabolic reprogramming limited DC maturation, migration, and chemokine production, including CXCL9 and CXCL10, thereby reducing recruitment and activation of cytotoxic T cells and weakening immune surveillance(PMID: 36891351).

Finally, IL-37 promoted tumor-permissive immune environments by modulating cytokine polarization (PMID: 30735937). Experimental models demonstrated that IL-37 suppressed sustained Th1-associated inflammatory cytokines, such as IFN-γ and TNF-α, while enhanced Th2-associated cytokines, including IL-4 and IL-13, as well as signals linked to alternative macrophage activation. Depending on the inflammatory context, this shift in immune balance may favor immune tolerance and tissue repair over effective tumor clearance (PMID: 30735937). Taken together, these findings demonstrate that IL-37 is not uniformly tumor-suppressive. Instead, its overall impact on cancer is context-dependent and determined by multiple factors, including the cellular source of IL-37, the immune composition of the tumor microenvironment, and the balance between direct tumor-inhibitory effects and suppression of immune surveillance.

## 7 Interplay of IL-37 with other interleukins

### 7.1 The modulatory role of IL-37 on IL-6 and IL-10 signaling

In the context of cancer, the interactions of IL-37 with IL-6 (46, 102, 103), and IL-10 (104) are particularly noteworthy due to their implications for tumor progression and immune regulation.

IL-37 plays a significant role in modulating cancer progression through its interactions with IL-6, impacting various cancers including lung adenocarcinoma (102), non-small cell lung cancer (NSCLC) (74), renal cell carcinoma (RCC) (46), and leukemia (103). In lung adenocarcinoma, IL-37 inhibited the Warburg effect—a metabolic shift towards glycolysis that is typical in tumors—by reducing the expression of PFKFB3, an enzyme crucial for glycolysis (102). This action diminishes glucose uptake and lactate production, thereby curbing tumor growth. Furthermore, IL-37 disrupted the IL-6/STAT3 signaling pathway, which is vital for tumor cell migration and invasion. By lowering IL-6 levels, IL-37 effectively prevented the activation of STAT3, which is critical for promoting metastasis (102). In NSCLC, IL-37’s antitumor activity is similarly connected to its effect on IL-6 (74). IL-37 decreased IL-6 levels, leading to reduced activation of STAT3, a transcription factor involved in driving EMT. This transition enhanced tumor cell invasiveness and migration. By inhibiting IL-6, IL-37 lowers the expression of EMT markers such as vimentin and N-cadherin, while increasing E-cadherin, thereby limiting tumor progression (74). In RCC, IL-37’s role in suppressing IL-6 signaling also contributes to its antitumor effects (46). Lower levels of IL-37 in RCC patients correlate with more advanced tumor stages. IL-37 reduced IL-6-induced STAT3 activation, which in turn decreased the expression of tumor-promoting factors like Bcl-2 and cyclin D1, thereby inhibiting tumor growth and progression (46). In leukemia, IL-37’s interaction with IL-6 is crucial for understanding its role in disease progression. Research has shown that IL-37 expression is downregulated in newly diagnosed acute myeloid leukemia (AML) patients but restored in those achieving complete remission. IL-37 negatively correlates with IL-6 levels, suggesting that IL-37 can suppress IL-6 production and its inflammatory effects in AML. By reducing IL-6 expression, IL-37 potentially limits the inflammatory environment that can contribute to leukemia progression, positioning IL-37 as an important factor in leukemia prognosis and therapy (103). In summary, IL-37 acts as a potent modulator of cancer through its regulation of IL-6.

Understanding how IL-37 interacts with IL-10 could provide insights into novel therapeutic strategies for managing diseases characterized by dysregulated inflammation and immune dysfunction (105, 106). Mountford et al. investigated whether IL-37 can offer protection against colon carcinogenesis in the context of chronic colitis (104). Using a mouse model, they crossed IL-37 transgenic (IL-37tg) mice with IL-10 deficient (IL-10KO) mice, which are prone to develop colitis and subsequently colon cancer. Both IL-10KO and IL-10KO/IL-37tg mice were subjected to mild colitis induced by dextran sulfate sodium (DSS) and then exposed to celecoxib to trigger colon cancer. They found that while both mouse groups experienced similar weight loss during the initial phase of colitis, IL-10KO/IL-37tg mice showed significantly better weight recovery after day 115. Analysis of inflammatory markers revealed that IL-37tg mice had lower levels of pro-inflammatory cytokines such as IL-6, IL-17, IFNγ, and TNFα, indicating reduced inflammation compared to IL-10KO mice. Histological examination showed fewer adenomas and no carcinomas in IL-10KO/IL-37tg mice, whereas six out of ten IL-10KO mice developed colon adenomas and carcinomas (104). Overall, IL-37 expression appears to protect against colon cancer during chronic colitis, likely by mitigating inflammation. However, it remains uncertain whether IL-37 directly suppresses tumor development.

### 7.2 Interplay of IL-37 with STAT-3

As mentioned above, IL-37 exerts its anti-inflammatory effects through a complex interaction with IL-18 receptor alpha (IL-18Ra) and IL-1 receptor 8 (IL-1R8). The interplay between IL-37 and STAT3 is a crucial aspect of this anti-inflammatory mechanism (107, 108). IL-37 binds to IL-18Ra, but its anti-inflammatory actions depend significantly on its association with IL-1R8 (also known as SIGIRR). This interaction forms a receptor complex involving IL-37, IL-18Ra, and IL-1R8, which is essential for IL-37’s function (108). Once the IL-37–IL-1R8 complex is formed, it activates a range of anti-inflammatory pathways. STAT3 (Signal Transducer and Activator of Transcription 3) plays a key role in this process (30). IL-37, through IL-1R8, promotes the activation of STAT3, which contributes to reducing inflammation by suppressing the production of pro-inflammatory cytokines and dampening inflammatory cell responses (109).

**Figure 2:**
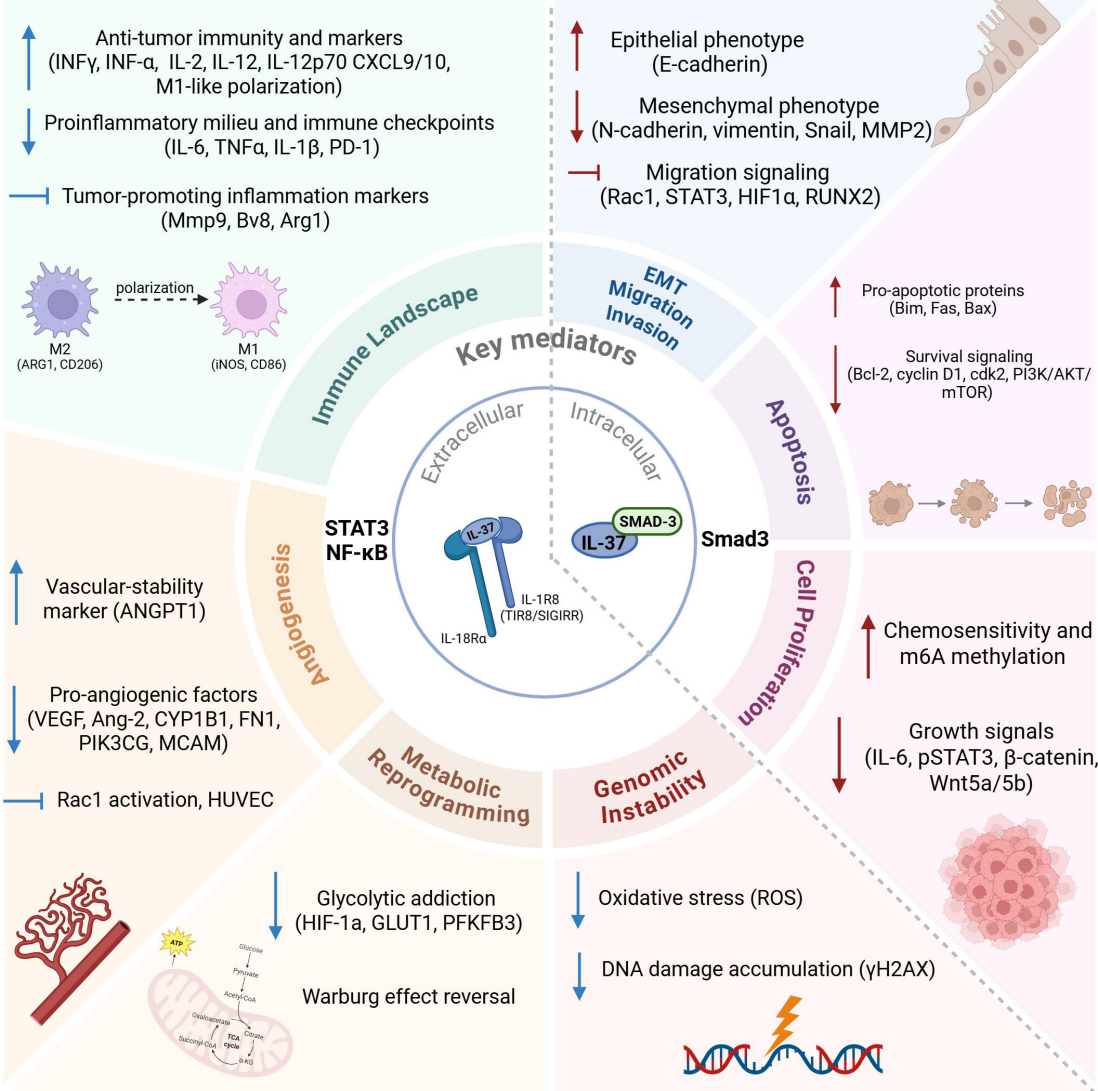
IL-37 modulates multiple cancer hallmarks via diverse molecular mechanisms. Mechanistic mapping of IL-37 influence on multiple cancer hallmarks. IL-37 serves as a central orchestrator of the tumor-suppressive landscape by intersecting with multiple cancer hallmarks via two signaling tracks_. The extracellular track (blue arrows) is initiated by IL-37 binding to the IL-1R8/IL-18Rα receptor complex, which suppresses STAT3 and NF-κB activity. This pathway attenuates tumor progression by remodeling the immune landscape, suppressing angiogenesis, reversing metabolic reprogramming, and maintaining genome stability. Conversely, the intracellular track (red arrows) involves mature IL-37 forming a complex with Smad3 and translocating to the nucleus. This pathway regulates gene expressions involved in reducing cell proliferation, enhancing apoptosis, and inhibiting migration/invasion/EMT. Overall, IL-37 upregulates anti-tumor effectors while downregulating pro-tumor mediators across the hallmarks of cancer. Created by BioRender https://BioRender.com/6kst0hy.

Wang et al. elucidated the role of IL-37 in regulating the STAT3 pathway in cervical cancer (CC) cells (47). They found that IL-37 gene transfection led to a substantial increase in IL-37 mRNA and protein levels in both HPV-positive Hela cells and HPV-negative C33A cells. This upregulation of IL-37 notably suppressed cell proliferation and invasion, with more pronounced effects in HPV-positive cells. Importantly, IL-37 expression was shown to inhibit STAT3. IL-37 reduced STAT3 expression at both mRNA and protein levels and decreased STAT3 phosphorylation, which is essential for its activation (47). Further investigation into the IL-37 and STAT3 interplay demonstrated that knocking down STAT3 with siRNA significantly diminished IL-37’s ability to inhibit cancer cell growth and invasion. On the other hand, overexpression of STAT3 reversed the inhibitory effects of IL-37, restoring cell proliferation and invasion to levels similar to those observed before IL-37 treatment. This restoration was accompanied by increased transcription of pro-inflammatory cytokines TNF-α and IL-1β, which IL-37 had previously suppressed (47). These findings highlight that the antitumor effects of IL-37 in cervical cancer cells are largely mediated through its inhibition of the STAT3 pathway, positioning IL-37 as a promising therapeutic agent targeting STAT3 for cervical cancer treatment. Finally, Zhang et al. engineered a vaccinia virus (VV) to express IL-37, creating a recombinant virus known as VV-IL-37 (48). They incorporated the IL-37 gene into the viral thymidine kinase (TK) region and tested its efficacy against HCC both *in vitro* and *in vivo*. The study involved infecting various HCC cell lines with VV-IL-37 and comparing the results to those from a control virus. The VV-IL-37 notably reduced cell viability and inhibited tumor cell proliferation, migration, and invasion. This was attributed to the enhanced expression of IL-37, which significantly lowered STAT3 phosphorylation, a key signaling pathway associated with tumor progression (48). Taking a step further, they demonstrated that VV-IL-37 not only promoted higher IL-37 levels in HCC cells but also effectively repressed tumor growth in a mouse model. The *in vivo* results showed smaller tumors and reduced STAT3 activity in animals treated with VV-IL-37 compared to those treated with the control virus (48). This study underscores the potential of VV-IL-37 as a powerful oncolytic virus capable of delivering IL-37 to suppress tumor growth and highlights its therapeutic promise in treating HCC by targeting the STAT3 signaling pathway. The effect of IL-37 in the different types of immune cells is illustrated in **Figure 2**.

## 8 Modulating CAR-T therapy with IL-37

Hamilton et al. investigated the impact of IL-37 on CAR T-cell therapy, particularly in the context of aging-associated immune decline (110). CAR T-cell therapy has shown promise in treating hematological malignancies like B-cell acute lymphoblastic leukemia (B-ALL). However, its efficacy can be compromised in older patients due to age-related immune dysfunction. They found that rhIL-37 treatment in aged mice led to significant improvements in the function of CAR T-cells. Aged CAR T-cells, typically exhibiting diminished potency, showed enhanced cytokine production, such as IL-2 and IFN-γ, after IL-37 treatment. This was evident in both *in vitro* and *in vivo* experiments. IL-37 treatment also resulted in a decrease in surface expression of PD-1, a marker associated with T-cell exhaustion, on both endogenous and CAR T-cells. By antagonizing TNF-α signaling and reducing NF-κB activation, IL-37 mitigated immunosuppressive mechanisms and promoted a more robust immune response. Furthermore, they demonstrated that IL-37 treatment not only rejuvenated endogenous T-cell functions but also enhanced the efficacy of aged CAR T-cells in combating B-ALL in murine models (110). These findings suggest IL-37 may improve CAR T-cell function in preclinical models of aging, although its impact on therapeutic outcomes in patients remains to be determined. Another study by Caraffa et al. explored the role of IL-37 in mitigating the inflammatory side effects associated with CAR-T cell therapy (111). CAR-T cell therapy can cause significant inflammation and toxicity due to the release of pro-inflammatory cytokines like IL-1 and IL-33, which activate mast cells and exacerbate the immune response (112). This inflammation can lead to serious adverse events such as fever, neurological toxicity, and organ damage. IL-37 has been shown to suppress the activity of pro-inflammatory cytokines, including IL-1 and IL-33. By binding to the IL-18 receptor alpha (IL-18Rα), IL-37 inhibited the production and activity of these cytokines, thereby reducing the inflammatory response. The study suggested that incorporating IL-37 into CAR-T cell therapy could potentially reduce the inflammation and toxicity associated with the treatment (111). This combination may have the potential to enhance the safety and efficacy of CAR-T cell therapy, pending validation in clinical studies.

## 9 Discussion

IL-37 has emerged as a key immunomodulatory cytokine with pleiotropic effects on both innate and adaptive immunity, exerting a broad influence on cancer-related processes. A number of shared mechanisms lie beneath its function across the various tumor types. First, IL-37 acts as a suppressor of chronic inflammation, primarily through inhibition of IL-6/STAT3 and NF-κB signaling pathways, thereby limiting tumor-promoting inflammation, epithelial–mesenchymal transition (EMT), and cancer cell proliferation. Notably, these pathways overlap with those driven by pro-inflammatory cytokines such as IL-17, which has been broadly implicated in cancer progression across multiple tumor types through activation of IL-6/STAT3, NF-κB, and other tumor-promoting signaling cascades (Begagić E, et al. The role of interleukin-17 in cancer: a systematic review, 2025). Recent systematic evidence further supports a predominantly pro-tumorigenic role for IL-17 in cancers such as breast, colorectal, and lung malignancies, where it correlates with poor clinical outcomes and enhanced tumor growth (Begagić E, et al.). In this context, IL-37 may function, at least in part, as a counter-regulatory cytokine that restrains IL-17–driven inflammatory tumor-promoting networks.

Second, IL-37 reorchestrates the tumor microenvironment through the reprogramming of immune cell subtypes, enhancing the polarization of macrophages towards the M1-like phenotype and promoting NK cells. Third, its highly context-dependent impact on adaptive immunity has the competence to support immune surveillance or, alternatively, dampen effective anti-tumor T cell responses. Altogether, the above mechanisms place IL-37 as a central regulator of the tuning between tumor-promoting inflammation and anti-tumor immunity.

Within the adaptive immune compartment, IL-37 preserves B cell progenitor integrity during aging by limiting inflammatory stress and preventing the emergence of oncogenic clones (100, 101). It also amends T cell responses in a context-dependent manner; restraining Th1-driven immunopathology in autoimmune hepatitis (92), while impairing CD8^+^ T cell activation and promoting tumorigenesis in colitis-associated cancer (93, 95). These data highlight a recurring effect across cancers: IL-37 can either maintain immune homeostasis or constrain effective anti-tumor immunity depending on the inflammatory milieu.

In innate immunity, IL-37 exerts consistent regulatory effects across tumor types by enhancing cytotoxic and anti-tumor functions and limiting pro-tumor inflammation. It augments the activity of NK cells via IL-1R8 downregulation and ERK/NF-κB signaling (76, 77), promotes M1 macrophage polarization via IL-6/STAT3 inhibition (82), and preserves neutrophil anti-tumor function by preventing C/EBPβ-driven pro-tumor polarization induced by circulating tumor cells (83, 86). However, its effects on dendritic cells remain context-dependent, through the enhancement of dendritic cell maturation and cytotoxic T lymphocyte activation in hepatocellular carcinoma (90), while impairing CD103^+^ dendritic cell metabolism and migration in skin squamous cell carcinoma, thus limiting CD8^+^ T cell responses (91).

At the molecular level, IL-37 broadly disrupts key tumor-promoting signaling networks. Through interactions with IL-6 and IL-10, it hinders STAT3 activation and downstream pathways linked to proliferation, angiogenesis, and immune evasion, as shown in lung adenocarcinoma, renal cell carcinoma, and leukemia (46, 74, 102, 103). Also, direct inhibition of STAT3 signaling by IL-37 reduces cancer cell proliferation and invasion, a mechanism that has been therapeutically examined using IL-37-expressing recombinant vaccinia virus models (47).

Collectively, these findings render IL-37 as a dual-edged regulator of tumorigenesis with impact on a limited number of core pathways—particularly inflammation control, immune cell reprogramming, and STAT3 signaling—while remaining highly dependent on tumor type, disease stage, and immune context. This duality positions IL-37 as both a potential therapeutic target and a biomarker of immune–tumor dynamics. The clinical significance of IL-37 as a prognostic indicator and its expression patterns across malignancies are summarized in **Table 5**.

**Table 5:** Clinical significance and expression patterns of IL-37 across human malignancies.

Tumor type	Reference (author, year)	Sample size (n)	Analyte (method)	Direction of association	Clinical endpoints & prognostic value
Hepatocellular Carcinoma (HCC)	Zhao et al. (77)	163	Tissue (IHC)	Lower in tumor	Correlates with larger tumor size, advanced TNM stage, and poor OS/DFS.
Colorectal Cancer (CRC)	Wang et al. (95)	32 patients (+21 controls)	Serum (ELISA)/Tissue (IHC)	Higher in patients	Positively correlates with CEA levels; negatively correlates with CD8^+^ T-cell infiltration.
Melanoma	Osborne et al. (96)	98 (49 patients + 49 controls)	Blood (qPCR)/Tregs (Flow cytometry)	Higher in blood	Exceptionally high expression in regulatory T cells (Tregs); driven by tumor-secreted TGF-β.
NSCLC (Intratumoral)	Ge et al. (61)	182	Tissue (IHC)	Lower in tumor	Independent predictor of poor OS; correlates with high microvessel density (MVD).
NSCLC (Systemic)	Jiang et al. (32)	80 (40 patients + 40 controls)	Plasma (ELISA)	Lower in plasma	Significantly correlates with advanced TNM stage (III–IV vs. I–II).
Renal Cell Carcinoma (RCC)	Jiang et al. (46)	170 (120 patients + 50 controls)	Serum (ELISA)	Lower in serum	Negatively correlates with tumor size and advanced TNM stage.
Colon Cancer	Yan et al. (50)	186	Tissue (IHC; qPCR; ELISA)	Lower in tumor	Correlates with lymph node metastasis, invasion depth, and poor survival; suppresses β-catenin signaling.
Oral Squamous Cell Carcinoma (OSCC)	Ding et al. (94)	42 patients (+22 controls)	Serum (ELISA)	Lower in serum	Lower IL-37/higher IL-18 ratio associated with advanced stage and poor OS.

IL-37 must also be interpreted within the broader cytokine network, including IL-1, IL-2, IL-6, IL-10, IL-12, and IL-15, which jointly orchestrate the tumor–immune interface (113). IL-2 directs T cell proliferation and cytotoxicity however is limited clinically by toxicity and short half-life, encouraging the development of next-generation variants with improved selectivity (114–122). IL-10, although classically immunosuppressive, can enhance CD8^+^ T cell responses in tumors, with pegylated IL-10 showing mixed clinical outcomes (112) (117–120),. IL-12 promotes Th1 and NK cell responses but requires targeted delivery strategies due to systemic toxicity (123–127). In contrast, IL-1 and IL-6 predominantly support tumor progression through NF-κB and STAT3 signaling, respectively, and are being actively targeted in clinical settings (128–136).

Regardless of the increasing interest in IL-37, its translational potential is constrained by several limitations. Much of the current evidence is derived from preclinical systems, including IL-37 transgenic models, which may not fully recapitulate human tumor immunobiology, especially given the absence of a murine homolog. Moreover, the dual and context-dependent nature of IL-37-ranging from anti-inflammatory and tumor -suppressive to immunosuppressive and tumor-promoting- confounds its therapeutic application. These effects are influenced by tumor type, stage, immune composition, and microbiota, limiting generalizability. Lastly, clinical data remain limited, and key parameters such as optimal delivery strategies, dosing, and safety profiles are not yet established. Future mechanistic studies and well-designed clinical trials are required to define how IL-37 can be effectively integrated into the cancer immunotherapy armamentarium.

## 10 Conclusion - future perspectives

Looking ahead, future research should aim to clarify the context-specific roles of IL-37 across different tumor types and immune environments. Advances in humanized mouse models and single-cell technologies may help unravel its cell-type–specific actions within the tumor microenvironment. Moreover, the development of targeted IL-37 delivery systems or engineered variants could enhance its therapeutic index while minimizing unwanted immunosuppression. To date, no interventional clinical trials have evaluated IL-37–based therapeutic strategies in cancer, which should be addressed with future studies. As our understanding deepens, IL-37 holds potential not only as a biomarker for immune modulation but also as a novel adjunct to existing immunotherapies. Well-designed clinical trials will be pivotal in translating its promise into clinical benefit.
